# *Wolbachia* Infections in *Anopheles gambiae* Cells: Transcriptomic Characterization of a Novel Host-Symbiont Interaction

**DOI:** 10.1371/journal.ppat.1001296

**Published:** 2011-02-17

**Authors:** Grant L. Hughes, Xiaoxia Ren, Jose L. Ramirez, Joyce M. Sakamoto, Jason A. Bailey, Anne E. Jedlicka, Jason L. Rasgon

**Affiliations:** 1 The W. Harry Feinstone Department of Molecular Microbiology and Immunology, Bloomberg School of Public Health, Johns Hopkins University, Baltimore, Maryland, United States of America; 2 The Johns Hopkins Malaria Research Institute, Baltimore, Maryland, United States of America; 3 The Institute for Genome Sciences, University of Maryland School of Medicine, Baltimore, Maryland, United States of America; ? Department of Epidemiology and Preventive Medicine, University of Maryland Baltimore, Baltimore, Maryland, United States of America; University of Ioannina, Greece

## Abstract

The endosymbiotic bacterium *Wolbachia* is being investigated as a potential control agent in several important vector insect species. Recent studies have shown that *Wolbachia* can protect the insect host against a wide variety of pathogens, resulting in reduced transmission of parasites and viruses. It has been proposed that compromised vector competence of *Wolbachia*-infected insects is due to up-regulation of the host innate immune system or metabolic competition. *Anopheles* mosquitoes, which transmit human malaria parasites, have never been found to harbor *Wolbachia* in nature. While transient somatic infections can be established in *Anopheles*, no stable artificially-transinfected *Anopheles* line has been developed despite numerous attempts. However, cultured *Anopheles* cells can be stably infected with multiple *Wolbachia* strains such as wAlbB from *Aedes albopictus*, wRi from *Drosophila simulans* and wMelPop from *Drosophila melanogaster*. Infected cell lines provide an amenable system to investigate *Wolbachia*-*Anopheles* interactions in the absence of an infected mosquito strain. We used Affymetrix GeneChip microarrays to investigate the effect of wAlbB and wRi infection on the transcriptome of cultured *Anopheles* Sua5B cells, and for a subset of genes used quantitative PCR to validate results in somatically-infected *Anopheles* mosquitoes. *Wolbachia* infection had a dramatic strain-specific effect on gene expression in this cell line, with almost 700 genes in total regulated representing a diverse array of functional classes. Very strikingly, infection resulted in a significant down-regulation of many immune, stress and detoxification-related transcripts. This is in stark contrast to the induction of immune genes observed in other insect hosts. We also identified genes that may be potentially involved in *Wolbachia*-induced reproductive and pathogenic phenotypes. Somatically-infected mosquitoes had similar responses to cultured cells. The data show that *Wolbachia* has a profound and unique effect on *Anopheles* gene expression in cultured cells, and has important implications for mechanistic understanding of *Wolbachia*-induced phenotypes and potential novel strategies to control malaria.

## Author Summary

*Wolbachia* are bacteria that infect many insect species, but do not infect *Anopheles* mosquitoes. These mosquitoes transmit *Plasmodium* parasites, which cause malaria in humans. *Wolbachia* infection in *Aedes aegypti* mosquitoes reduces their ability to transmit diverse pathogens including viruses, nematodes and bird malaria parasites. *Wolbachia*-induced stimulation of the mosquito's innate immune system has been suggested as a mechanism conferring this pathogen interference. Since no *Wolbachia*-infected *Anopheles* mosquito strain exists, we used infected cultured *Anopheles* cells to examine the effect of infection on *Anopheles* gene expression. *Wolbachia* had a profound influence on *Anopheles* gene expression. Many of the genes regulated by *Wolbachia* have been seen in other studies to influence *Plasmodium* levels in mosquitoes, but interestingly and in contrast to other mosquitoes, many of the host genes were suppressed rather than induced.

## Introduction

*Wolbachia* are alpha-proteobacteria that infect a range of arthropods and nematodes, and are possibly the most common endosymbiotic bacteria on the planet. In their arthropod hosts, *Wolbachia* induce a variety of reproductive manipulations that enhance the fitness of infected females compared to their uninfected counterparts [1]. *Wolbachia* have recently been shown to interfere with pathogen infection and transmission in both naturally-infected and artificially-transinfected insects [2], [3], [4], [5], [6], [7]. These phenotypes make *Wolbachia*-based control strategies an attractive option to minimize the impact of arthropod-borne diseases and insect pests [8], [9].

*Anopheles* mosquitoes transmit human malaria, a devastating disease that kills approximately 2 million people per year, and are naturally uninfected with *Wolbachia* [10], [11], [12]. Transfer of *Wolbachia* into cultured *Anopheles gambiae* cells and transient somatic infection of adult female mosquitoes demonstrates that the bacteria can survive in this species, suggesting that the *Anopheles* genus may be amenable to stable infection [13], [14]. Although several novel *Wolbachia*-mosquito associations have been created using a variety of transinfection techniques, no stable *Wolbachia*-infected *Anopheles* line has been developed [15], [16], [17], [18], [19], [20]. The development of such a strain may open the possibility for *Wolbachia*-based control strategies for malaria. Indeed somatic infections of the wMelPop strain reduce oocyst levels in the murine malaria model, *Plasmodium berghei* [7]. However the global effects of *Wolbachia* on *Anopheles* and the interplay within the tripartite association of the human malaria *Plasmodium* parasites and the mosquito host are currently unknown.

Novel phenotypes are sometimes observed upon transinfection of *Wolbachia* into novel insect hosts [15], [21], [22]. In the artificially infected wMelPop-*Aedes aegypti* strain (wMelPop CLA), *Wolbachia* limits infection by a broad range of pathogens including dengue virus, filarial nematodes and *Plasmodium* [2], [3]. The mode of action for pathogen resistance is uncertain, however two mechanisms have been postulated; immune activation of the host by *Wolbachia* and/or metabolic competition between the bacteria and the pathogen. Evidence for both hypotheses was observed with a range of immune genes up-regulated in wMelPop-infected *Ae. aegypti* [2], [3] and the finding that dengue virus only persisted in *Wolbachia*-uninfected cells of the insect [3]. A similar phenotype was observed in some infected *Drosophila* strains where *Wolbachia* infection induced refractoriness to multiple RNA viruses [4], [5]. Interestingly, a previous study using naturally infected hosts found that *Wolbachia* seems to be able to evade the host immune response in *Drosophila* and *Aedes albopictus* [23], suggesting *Wolbachia*-induced immune activation may be more likely in novel rather than co-evolved *Wolbachia*-host associations.

Within *Anopheles* mosquitoes, there is a conserved immune response towards foreign bacteria and *Plasmodium* [24]. By using multiple methods such as co-feeding, injection or removal of microflora, bacteria have been seen to mediate *Plasmodium* infection levels in the *Anopheles* host [25], [26], [27], [28], which is thought to be due to the bacteria priming the host immune response. Interestingly, Gram-negative bacteria elicit a greater response compared to Gram positive, although there are species-specific differences [25], [27]. If *Wolbachi*a (a Gram-negative bacterium) evokes a similar response and up-regulates the basal immunity in infected *Anopheles*, infection may confer an anti-*Plasmodium* phenotype. Some evidence for this has been shown in somatically-infected mosquitoes infected with rodent malaria [7].

The generation of *Wolbachia*-infected *Anopheles* cell lines allows the investigation of *Wolbachia*-*Anopheles* interactions in the absence of a stably-infected mosquito strain [14]. Cell lines provide a platform whereby *Wolbachia* host lineages can be generated with relative ease, and allow the exploration of both natural and artificial *Wolbachia* host interactions [29], [30], [31]. To investigate the effect of *Wolbachia* infection on global patterns of *Anopheles* gene expression we performed microarray analysis on both wAlbB (from *Ae. albopictus*) and wRi (from *Drosophila simulans*) infected *Anopheles gambiae* Sua5B cells compared to uninfected cells. We validated microarray results *in vitro*, *and in vivo* for a subset of differentially expressed genes in somatically-infected adult female mosquitoes.

## Results/Discussion

**Figure 1:**
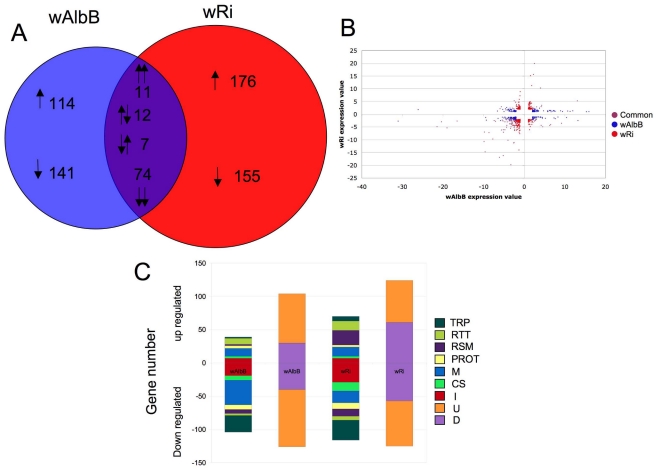
*Anopheles gambiae* gene regulation in response to *Wolbachia* infection. A. Venn diagram of 690 *Anopheles* transcripts which display differential expression due to wAlbB or wRi infection. 104 transcripts were common to both strains, while 389 were down regulated and 320 up regulated due to *Wolbachia* infection. B. Scatter plot of regulated significant genes (>2 fold regulation; False discovery rate P value <0.05). Blue dots represent significant genes regulated by wRi only, red regulated by wAlbB only and purple, genes commonly regulated. C. Number of genes in each functional classes class up or down regulated in response to either wAlbB or wRi infection. Genes were classified into groups; transport (TRP), replication, transcription and translation (RTT), redox, stress and mitochondrial (RSM) proteolysis and digestion (PROT), metabolism (M) cytoskeletal and structural (CS) and immune (I) depicted in the first column, and diverse (D) and unknown (U), in the second column.

*Wolbachia* infection of *Anopheles* cells resulted in the regulation of 690 genes relative to uninfected Sua5B cells (False discovery rate (FDR) P<0.05, ≥ 2.0 fold-change (FC)) (Table S1). When comparing *Wolbachia* strains, 255 genes were uniquely regulated by wAlbB infection, while 331 were regulated specifically by wRi infection (Figure 1A). Of the 104 genes regulated by both strains, the majority (74 genes) were down-regulated, 11 were similarly up-regulated and the remainder had alternating regulation patterns between the two *Wolbachia* strains (Figure 1B). Interestingly, we observed a greater number of genes regulated by wRi compared to wAlbB even though the cell infection density of wRi was much less than wAlbB (wRi∼10% cells infected, wAlbB >90% of cells infected) [14]. It is possible that since wRi was purified from live flies, it has a greater impact that wAlbB which was purified from another cell line [14]. Of the regulated genes, a diverse range of functional classes was represented with a large proportion being genes of unknown or diverse function, which was consistent for both *Wolbachia* strains. Among the genes assigned to specific known functional classes, immune-, transport- and metabolism-related transcripts were the most abundant categories regulated by *Wolbachia* (Figure 1C). Strikingly, over 75% of the immune related transcripts were down-regulated, which was consistent between both strains. Overall, down-regulation was a common theme, with only redox/stess/mitochondrial (RSM) and replication/transcription/translation (RTT) classes not down-regulated in wRi infected cells and RTT in wAlbB. Microarray data is available at gene expression omnibus (accession number GSE23215) [32].

### qPCR validation of microarray genes and comparison to whole mosquitoes

**Figure 2:**
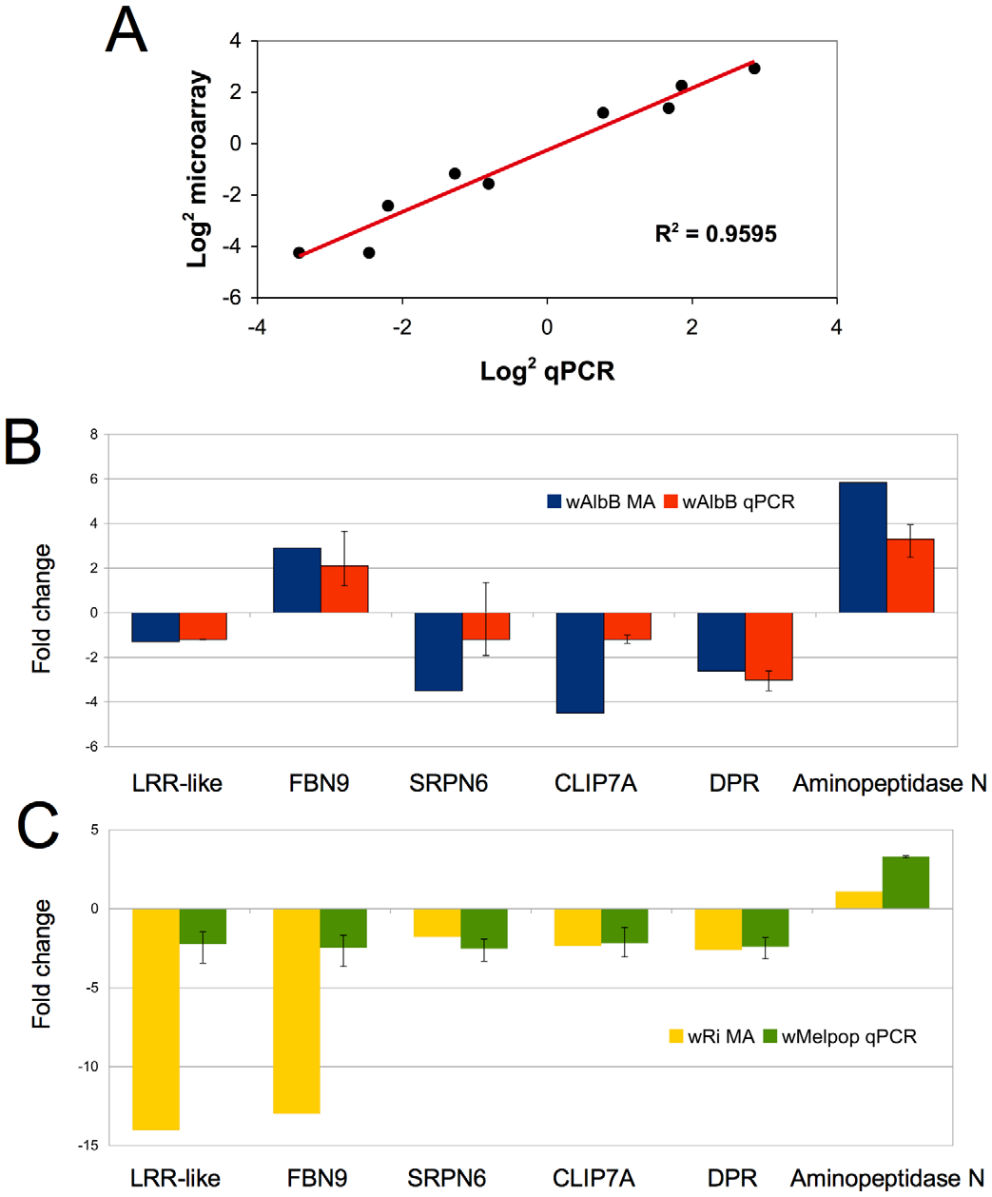
Validation of microarray data in cell culture and whole mosquitoes. A. Log2 fold change for selected *An. gambiae* genes (HSP20, HSP90, HSPDnaJ, cold-shock protein, cecropin, Serpin6, Filamin, TEP3) comparing microarray and QPCR methods. B. Comparison of *Anopheles* gene expression in response to *Wolbachia* in cell culture and whole mosquitoes. Expression of 6 genes from wAlbB in Sua5B cells analyzed using microarrays (MA) compared to wAlbB somatically-infected whole mosquitoes 15 days post injection (N = 5 mosquitoes/treatment). C. Microarray data from wRi infected Sua5B cells compared to wMelpop somatically-infected whole mosquitoes 15 days post injection (N = 5 mosquitoes/treatment). qPCR gene expression is a ratio of *Wolbachia* infected (wAlbB or wRi) to Schneider's injected control. Error bars represent maximum and minimum range of expression.

To gauge the accuracy of the microarray data, we selected a subset of genes to validate by quantitative real-time PCR from cell culture. Eight genes, (HSP20, HSP90, HSPDnaJ, cold-shock protein, cecropin, Serpin11, Filamin, TEP3) with varying expression profiles, regulated by both *Wolbachia* strains were evaluated. These genes spanned a variety of functional classes including defensive and immune genes that may be relevant to *Plasmodium* infection and potential *Wolbachia*-mediated reproductive phenotypes. qPCR results corroborated the array data and had a positive linear correlation (R^2^ = 0.9595) when comparing the log2 values using both gene expression techniques (Figure 2A).

*Wolbachia* has been shown to persist, disseminate, and replicate in injected adult *Anopheles* mosquitoes [13]. We injected live female mosquitoes with *Wolbachia* to determine if the effect of infection on gene regulation *in vivo* was consistent with results observed from infected cell cultures. Several immune related transcripts and other genes, which potentially convey interesting phenotypes and had varying expression profiles identified in cell culture, were assessed. When comparing wAlbB regulation in cells and mosquitoes, the direction of regulated expression was similar (Figure 2B), although, not surprisingly, the intensity of expression varied leading to a lack of significant correlation (data not shown). The loss of the wRi-infected cell line prevented a direct comparison to somatically infected mosquitoes, however, this array data was compared to wMelPop-infected mosquitoes. wMelpop and wRi both infect *Drosophila* and are classed in supergroup A. When making this comparison, again we observed that the direction of gene regulation was similar (Figure 2C), but the intensity of expression varied. Notably, the intensity of two genes, the LRR-like transcript and FBN9 is greater in the cells compared to the whole mosquito. This may be explained by the hemocyte-like character of the cell line or *Wolbachia* strain-specific variation. Nevertheless, the similarity in the direction of gene regulation *in vivo* and *in vitro* suggests that the effect of *Wolbachia* in the cell line may be applicable to whole mosquitoes.

### Comparison to other systems

We compared *Wolbachia*-regulated *Anopheles* transcripts identified in this study to genes regulated by wMelPop in *Aedes aegypti* [2] and by bacterial infection in *A. gambiae* [26]. Fourteen *A. aegypti* homologues were identified from differentially expressed *Anopheles* transcripts in response to *Wolbachia* infection, with five having an immune related function (Table S2). When comparing these results, 75% of both wRi and wAlbB regulated homologs displayed a similar direction of expression. Similarly, when comparing *Wolbachia*-regulated transcripts to those of regulated by bacterial infection in *A. gambiae*, 15 homologs were regulated by *Wolbachia*. (Table S3). Most of these homologs were of unknown function.

In comparison to other studies using *Drosophila* cell culture systems to examine the influence of *Wolbachia* on host gene expression, we find a dramatically elevated number of identified regulated *Anopheles* genes compared to *Drosophila*. In *Drosophila* S2 cells, 263 genes had a 1.2 fold change due to *Wolbachia* infection, however when the more common ≥2 fold criteria was used, very few regulated *Drosophila* genes were identified [33]. At the proteomic level, only four proteins, all host antioxidant proteins, were elevated in *Wolbachia* infected *Ae. albopictus* Aa23 cells [34]. A lower *Wolbachia* titer may account for the subtle gene regulation in wRi infected *Drosophila* cells [33], although the infection density of wRi in infected *Anopheles* was similarly sparse [14]. Alternatively, the mild effect of *Wolbachia* on gene regulation in *Drosophila* and protein expression in *Ae. albopictus* could be due to previous co-evolution between the *Wolbachia* strains and their naturally infected hosts.

### Effect of *Wolbachia* on transcription of *Anopheles* genes potentially affecting pathogen transmission

#### Stress-response

The most striking effect observed for both wRi and wAlbB infections was the general suppression of heat shock protein transcripts (HSP20, HSP70, HSP90, HSP-DNAJ). Cells infected with wAlbB had a dramatic suppression of these genes with 5 out of the top 6 most down-regulated genes (FC −31 to −16). Similarly, these genes were down-regulated by wRi, albeit to a lesser extent (to −5.3). Presenting a similar pattern of regulation, multiple HSPs were down-regulated by wRi infected *Drosophila* S2 cells [33]. *In vivo*, it has been shown that *Wolbachia*-infected flies have altered expression of HSP, which in turn affects *Wolbachia*-induced reproductive phenotypes [35]. HSPs have also been implicated in *Anopheles*-pathogen interactions. Elevated levels of HSP20 were identified in *An. gambiae* heads after infection with *P. berghei* [36]. If this protein assists transmission, either directly or indirectly, the antagonistic actions of may potentially reduce *P. berghei* sporozoite infection. Additionally, knockdown of a heat shock proteins (HSC70B) via injection of dsRNAi in conjunction with O'nyong nyong virus (ONNV) significantly reduced the lifespan of adult mosquitoes as compared with the control [37]. We speculate that if this expression pattern translates to *in vivo Anopheles* infections, *Wolbachia-*induced down-regulation of HSPs may modulate vector competence of ONNV or shorten mosquito lifespan.

#### Metabolic and other genes

*Wolbachia* regulates a suite of genes involved in *Anopheles* metabolism, with most of these transcripts being down-regulated by infection. Although the heterotrophic needs of *Plasmodium* and mosquito growth factors required for parasite development are not well understood in the insect, changes in transcription of metabolism genes which alter the mosquito environment may affect *Plasmodium* growth. Infection of Sua5B cells with wAlbB drastically reduces phosphoenolpyruvate carboxykinase (PEPCK) transcripts 26 fold. In response to *P. falciparum*, PEPCK is up-regulated in the mosquito [38], [39]. Carbonic anhydrase, which catalyses the reversible hydration of carbon dioxide to bicarbonate, is down-regulated in wAlbB-infected cells by 2.6 fold. In many mosquitoes, inhibition of this enzyme results in a reduction in pH of the mosquito midgut [40]. Moreover, carbonic anhydrase inhibitors in *P. falciparum* reduced parasite survival in the human blood stages and have been suggested as targets of anti-malarial drug design [41], [42]. The effect of these host derived enzymes on parasite development is unknown, however changes in regulation between mosquito and *Plasmodium* suggest that further examination of these genes is warranted to determine their affects on parasite development. Although not strictly metabolism related, laminin and collagen are components of the basal lamina, which are interrelated with parasite invasion [43], [44], [45]. Both laminin (FC −2.1, −3.8) and collagen (FC −4.4) are down-regulated by wRI infection. RNAi knock down of laminin lead to a substantial reduction of oocysts in mosquito midguts [44] possibly due to laminin inhibiting the melanotic encapsulation of oocysts [46].

#### Immunity-related transcripts

**Figure 3:**
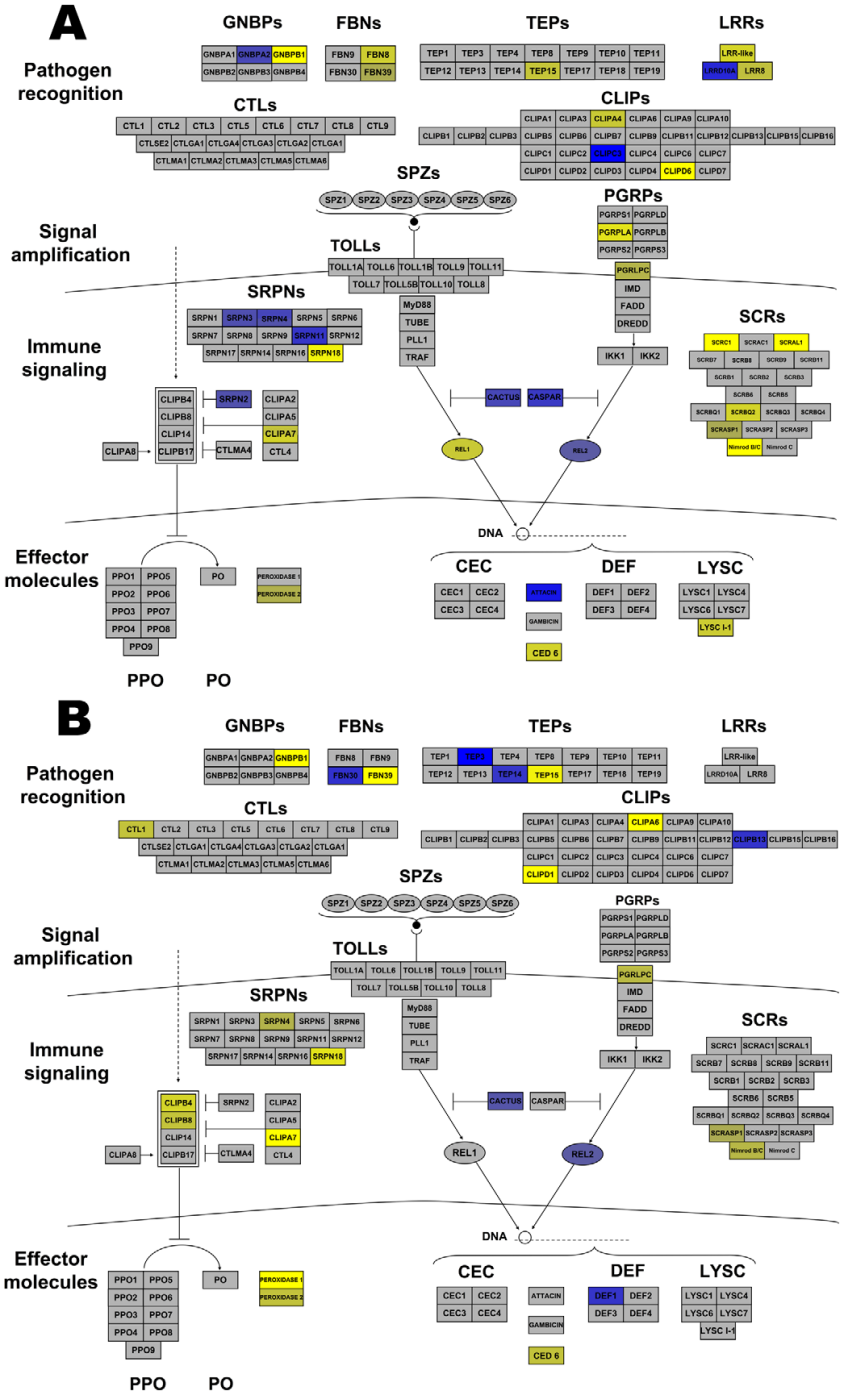
*Wolbachia* strain-specific regulation of *Anopheles gambiae* immune pathways. *Anopheles* immune networks regulated by wRi (A) and wAlbB (B). Pathways are models of the IMD and Toll pathways [81] and components of the melanization regulatory module [51] divided into the 4 broad categories of immune molecules. Blue color represents induction, while yellow color represents suppression. The intensity of coloring is proportional to the intensity of expression. Regulation is depicted to a maximum fold change of ±4. Some transcripts were greater than ±4 regulated. Abbreviations: LLR leucine rich repeats; FBNs fibrinogens; TEPs thioester containing proteins; GNBPs Gram-negative binding proteins; CTLs C type lectins; CLIPs clip-domain serine protease; PGRPs peptidoglycan recognition proteins; SRPNs serpins; CEC cecropins; Def defensins; PPO Prophenoloxidase; PO phenoloxidase; LYS lysozmyes.

Many *Anopheles* genes associated with arthropod immunity were regulated by *Wolbachia* infection. Genes within all the broad categories of immunity (pathogen recognition receptors, signaling amplification cascades, immune signaling pathways, and effector molecules) were regulated. Immune genes up-regulated by both infections included CLIPs and antimicrobial peptides (AMP), while serpins (SRPN), and a leucine rich repeat (LRR) were induced by wRi and fibrinogens (FBN) and thioester-containing protein (TEP) were induced by wAlbB (Figure 3). More striking were those immune genes down-regulated by infection. wRi significantly suppressed expression of class C scavenger receptors, Gram-negative binding proteins (GNBP), FBN, CLIP, SRPN, LRR-containing genes, a TEP, effector proteins involved in phagocytosis and a lysozyme (Figure 3). The wAlbB strain down-regulated genes of similar functions, however in the class of effector molecules, this strain had more of an influence on peroxidases rather than AMPs (Figure 3).

In addition, other immune-associated apoptosis and detoxification transcripts were regulated by infection. Brennan et al. [34] identified *Wolbachia*-induced host antioxidant proteins in cell culture. In contrast to the enrichment of these genes at the protein level, a peroxiredoxin transcript was down-regulated 2.1 times by wAlbB and 11 times by wRi. Likewise, superoxide dismutase was down-regulated in wRi-infected cells (FC −2). Additionally, eight glutathione S transferases were regulated. Two of these were co-regulated by both strains, while 3 were induced and 3 suppressed in wRi. The level of regulation for these genes was approximately 2–3 fold, however one transcript was suppressed 19-fold by wRi compared to uninfected cells. Taken in total, these data suggest that *Wolbachia* can significantly affect cellular defense, detoxification and immunity in *An. gambiae* cells, and that expression of many of these defensive genes is suppressed rather than induced. These results contrast with observations of up-regulation of the majority of immune-related transcripts in stably-infected *Ae. aegypti* mosquitoes, which have reduced capacity to transmit pathogens [2], [3]. Gene expression of a small subset of immune genes were characterized in response to wMelPop infection of a different *An. gambiae* cell line (Mos55), where they were up-regulated, suggesting a potential difference between *Anopheles* cell lines or *Wolbachia* strain-specific variation [7].

Although pathogen interference occurs in naturally infected hosts, there is evidence that the transfer of *Wolbachia* to a new host is a catalyst for pathogen interference, illustrated by wAlbB inducing dengue resistance in a novel host, *Ae. aegypti*, yet not conferring interference in it's native host, *Ae. albopictus* [6]. The effects of tripartite relationship of *Wolbachia*-*Anopheles-Plasmodium* are relatively unknown, however, recently wMelPop somatically infected into *Anopheles* was seen to decrease *P. berghei* oocyst levels, with evidence that TEP1 may involved in the process [7]. Many of the regulated defensive genes we identified have been shown to directly or indirectly affect *Plasmodium* infection in *Anopheles*, either positively or negatively. TEP3 was dramatically up-regulated (FC 7.6) in response to wAlbB. Similar up-regulation is observed when mosquitoes are fed a blood meal, either uninfected or infected (*P. berghei*), or challenged with bacteria [47], [48]. TEP1, a protein similar to TEP3, has been shown to be an important molecule involved in the melanization and anti-*Plasmodium* response across the *Anopheles* genus [49], [50]. Looking at genes involved in the immune signaling cascade, CLIP7A, a suppressor of melanization, was suppressed by both wAlbB (FC −5.2) and wRi (FC −2.6), which may confer an anti-*Plasmodium* phenotype as seen in knock-down experiments of this gene [51]. In contrast, the gene galectin, which is up-regulated in response to *P. berghei* infection and immune challenge by *Micrococcus luteus*, had conflicting strain-specific responses: up-regulated by wAlbB (FC 9.1) but down-regulated by wRi (FC −3.5) [52].

In contrast to genes that may abate *Plasmodium* infection, a suite of genes were also regulated in ways that may elevate parasite levels in infected mosquitoes. For example we observed down-regulation of many CLIPs. Reverse genetic techniques have shown that both CLIPB4 and CLIPB8 are involved in the melanization process, where knock-down of these genes ablates melanization [53]. In double knock-down (KD) experiments, reducing transcripts of both CLIPB4 and CLIPB8 in tandem with CTL4 partially interferes with *P. berghei* ookinete melanization [51]. Using over-expression, up-regulation of cecropin was shown to decrease *Plasmodium* levels in *Anopheles* [46]. Expression of both SRPN18 (FC wAlbB −3.2, wRi −3.6) and TEP15 (FC wAlbB −3.5, wRi −2.1) is suppressed by both *Wolbachia* strains and although the specific function of these molecules has not been identified, these classes of molecules are associated with immunity [48], [54]. In *Ae. aegypti*, TEP15 is one of the most strongly induced genes in response to KD of Cactus, the negative regulator of the Toll pathway [55]. In addition, GNBPB1, which was also down-regulated by both strains (FC wAlbB −5.2, wRi −6.0), is strongly induced by parasite invasion of the midgut and bacterial challenge [52], [56], [57]. In contrast to our study, GNBP was induced in *Aedes* mosquitoes infected with wAlbB and wMelpop [2], [6].

In terms of a general response to bacterial infection, we see the regulatory transcriptional factor for the Toll pathway (Rel1) down-regulated 2.3 times by wRi infection. We observed an up-regulation of caspar (FC 2.2), the negative regulator of the IMD pathway in response to wRi. PGRP-LA expression was suppressed 3.2 times by wRi. In *Drosophila*, PGRP-LA is likely to be a hemocyte transmembrane protein [58], while other PGRPs activate negative feedback loops in the IMD pathway [59], [60]. A similar long transcript PGRP (PGRP-LC) in *An. gambiae* controlled proliferation of gut microbiota, which subsequently influenced *Plasmodium* infection [61]. When all three PGRP-LC isoforms were silenced simultaneously, mosquitoes challenged with *Staphylococcus aureus* had induced expression of cecropin and defensin. In *Drosophila*, silencing of PGRP-LC by RNAi induced expression of diptericin, cecropin A1, and attacin A, but these effector molecules were not regulated due to depletion of PGRP-LA [62]. Here we see similar independent regulation of attacin which was up-regulated 3.3 times in wRi infected cells, while defensin is also up-regulated by wAlbB (FC 2.3). Interestingly, attacin was found to inhibit the outer membrane synthesis of *Escherichia* coli in the giant silk moth, *Hyalophora cecropia* [63]. Thus, we may be observing an active defensive response from *Anopheles* to prevent *Wolbachia* infection.

The general pattern of immune gene down-regulation appears to be a *Wolbachia*-specific phenomenon in this cell line. In addition to *Wolbachia*, Sua5B cells can support infection of additional intracellular bacteria such as *Rickettsia* [64]. We used qPCR to test selected immune-related genes (cecropin1, defensin1, gambicin and immune-responsive serpin-related protein [IserpF1]) in Sua5B cells that had been infected with a taxonomically and phenotypically diverse array of *Rickettsia* species: *R. typhi* (typhus group), *R. felis* (transitional group), *R. montenensis* and *R. peakockii* (both in the spotted fever group). *R. typhi* and *R. felis* are human pathogens, while *R. montenensis* is non-pathogenic. *R. peakockii* is a non-pathogenic vertically-transmitted tick endosymbiont. While there was variation between bacterial species and the gene tested, all four *Rickettsia* induced expression of most tested immune genes (up to 12-fold induction), including the endosymbiont *R. peakockii* (Figure S1). These results suggest that the natural response of Sua5B cells to intracellular bacterial infection is immune up-regulation, and that *Wolbachia* is suppressing this response. It should be noted however that *Wolbachia* exist in a potentially protective host vacuole, while *Rickettsia* are free in the cytoplasm.

### *Wolbachia* influence on reproduction-related genes

*Wolbachia*-induced CI expression is associated with abnormal decondensation of the paternal pronucleus during fertilization, epigenetic factors, and/or problems during embryogenesis. Xi et al. [33] observed that in wRi-infected *Drosophila* cells, the gene angiotensin converting enzyme (*Ance*), which is involved in spermatogenesis, was up-regulated by *Wolbachia* infection in cells and flies, and was potentially involved in the CI phenotype. In our study, the six *Anopheles* homologues of *Ance* on the microarray were not affected by *Wolbachia* infection. We screened our data for other significantly regulated genes associated with cytoskeleton formation/function, epigenetic modification, gametogenesis or embryonic development. Multiple cytoskeleton-associated genes, genes associated with chromatin formation and remodeling and genes associated with embryogenesis and cell division were regulated by both infections.

We identified multiple genes that may be linked to the CI phenotype. Transcription of a Kazal-like serpin was enhanced dramatically due to *Wolbachia* infection (FC wAlbB 13.1, wRi 5.3). Kazal domain-containing proteins identified in animals have a diverse array of functions. A Kazal-like serpin was found to inhibit both gelatinolytic activity of sperm and the proteolytic activity of sperm extracts to vitelline coat in prawns [65], while in mice, a serine protease inhibitor Kazal-type-like protein bound to sperm, enhancing motility and suppressing sperm capacitation [66]. Although in these two species the function of the Kazel-like serpin is varied, it has the commonality that it interferes with sperm–oocyte interactions. Up-regulated (FC 2.3) in wRi-infected cells, crooked neck (crn) transcripts are involved in embryogenesis. In it's recessive form, crn is lethal to embryos, while heterozygotes display a crooked phenotype [67]. In both *Drosophila* and humans, crn has been implicated in the mRNA splicing process and is thought to be a pre-mRNA splicing factor [68], [69]. Another gene induced by wRi (FC 3.1), otefin, codes for a nuclear laminin which is essential for germ cell maintenance in *Drosophila* [70]. A further candidate protein, Dumpy-30 (Dpy-30) is expressed in spermatids in *Drosophila*, and mutations or knockout of the male-specific *dpy-30L2* gene results in male sterility as mutant sperm have impaired motility and fail to accumulate in sperm storage organs of females [71]. In *Anopheles* cells, wAlbB up-regulates (FC 2.0; significant at unadjusted P<0.05) Dpy-30, and although the effect of over-expression is unknown, this could potentially have a role in the CI phenotype. Serine active site containing (Serac1) mediates sterility in mice [69] and is up regulated (FC 2.4) by wRi infection. TEP15, suppressed by both strains (FC wAlbB −3.5, wRi −2.2), may influence reproduction. TEP15 is a male accessory glands protein and is transferred to female in the mating plug [72]. It would be interesting to determine if *Wolbachia*-induced regulation of these transcripts is *Anopheles* specific or common to other insect species infected with *Wolbachia*.

In addition to these genes, heat shock proteins were dramatically down regulated by both bacterial strains, but the effect was most dramatic by wAlbB. HSPs have been associated with sperm production and are inferred to be involved in CI [35], [73]. A range of chaperone proteins were also up-regulated by wRi, including a cold shock protein (FC 4.8) multiple DNAJ heat shock proteins (FC 3.3, 2.1), GrpE protein (FC 2.7), and a ubiquilin-1 gene (FC 2.3).

### Pathogen related phenotypes

Other identified regulated genes may have behavioral implications for infected *Anopheles*. It has been reported that some older wMelPop-infected *Ae. aegypti* mosquitoes have “bendy” and “shaky” phenotypes [74], [75]. The proboscis of “bendy” individuals is flexible and unable to penetrate the skin [74]. Mosquitoes with the “shaky” phenotype have a jittering action of the insect body [75]. Here, we have identified genes that may elucidate these phenotypes at the molecular level. Both *Wolbachia* strains suppress the defective proboscis extension response (dpr) gene (wAlbB −3.3, wRi −2.6). Moreover, this gene is also down regulated in *Wolbachia-*injected mosquitoes (wAlbB −3.0, wMelPop −2.4; Figure 2B & 2C). In *Drosophila*, dpr is part of a gene family encoding predicted cell adhesion molecules that contain two Ig domains [76]. It is possible that a reduction in cell adhesion causes plasticity in the proboscis leading to the “bendy” phenotype. In addition to reduced dpr transcripts, *Wolbachia* down-regulated numerous other cell adhesion genes. Interestingly, dpr also has been shown to be required for the proper timing of male courtship [76], and given that *Anopheles* have elaborate swarming courtship behaviors in the wild, *Wolbachia* infection may have the potential to alter reproductive success.

Sestrins (Sesn), a family of conserved proteins, accumulate in cells in response to stress and are inhibitors of target of rampamycin (TOR) that prevent age-related pathologies [77], [78]. In wAlbB-infected cells, we see a down regulation of Sesn (FC −3.5). In *Drosophila* dSesn-null mutants, age related degeneration of muscle was observed in the form of cardiac malfunction and abnormal skeletal muscle [78]. Possibly, suppression of Sesn in wMelpop infected *Ae. aegypti* is related to the “shaky” phenotype [75]. Moreover, it would be interesting to correlate Sesn levels in both *Drosophila* and *Ae. aegypti* infected with wMelpop, which display life shortening and age related pathologies [17], [79], to determine if Sesn plays a role in life shortening from this strain of *Wolbachia*. The “shaky” and “bendy” phenotypes are more prevalent in older *Wolbachia* infected *Aedes* mosquitoes [75]. If the genes identified here confer the “bendy” and “shaky phenotypes in a *Wolbachia*-infected *Anopheles* mosquito, these effects could be more influential on malaria transmission compared to direct pathogen interference.

### Conclusion

*Wolbachia*-infected mosquito cells provide a tractable platform to characterize *Wolbachia-Anopheles* transcriptomic interactions in the absence of a stably-infected mosquito strain. Using this system, we identified a suite of *Anopheles* genes regulated by two divergent *Wolbachia* strains. As a general theme, *Wolbachia* have a profound effect on transcription of many host defensive genes, possibly to facilitate and maintain intracellular infection. These data may give insights into the transfer of *Wolbachia* into novel hosts, *Anopheles-Wolbachia* interplay, interaction with pathogens transmitted by *Anopheles* and other *Wolbachia*-induced phenotypes such as reproductive manipulations.

## Materials and Methods

### Cell culture

*Wolbachia*-infected (wRi and wAlbB) and uninfected Sua5B cells were generated and cultured as previously described [14]. Both cell lines were >30 passages post-infection at the time of experiments. Cell line transcriptome expression was assessed using the Affymetrix *Anopheles/Plasmodium* GeneChip. Processing of samples for microarray analysis was performed by the Johns Hopkins Malaria Research Institute Gene Array Core Facility (JHMRI-GACF), using standard Core protocols as described below.

### RNA extraction

Cells were harvested, washed, resuspended in PBS, flash frozen in liquid nitrogen, and stored at −80°C. Homogenization and lysis of cells was performed with Lysing Matrix D (Qbiogene) in Trizol LS reagent (Invitrogen) by rapid agitation in a FastPrep 120 Instrument (Qbiogene) for 15 seconds at speed setting 5. Homogenates were subsequently processed according to the manufacturer's (Invitrogen) protocol with the following minor modifications. Two microliters of 5 mg/ml glycogen was used as a carrier for overnight isopropanol precipitation, and all centrifugation times were increased to 15 minutes. RNA pellets were resuspended in Nuclease-free water. Further purification was performed using the Qiagen RNeasy Mini kit, according to manufacturer's recommended protocol. Quantitation of RNA was performed using a NanoDrop spectrophotometer, and quality assessment determined by RNA Nano LabChip analysis on an Agilent BioAnalyzer 2100.

### Affymetrix GeneChip protocols

Processing of templates for GeneChip Analysis was in accordance with methods described in the Affymetrix GeneChip Expression Analysis Technical Manual, Revision 5. Double stranded cDNA was synthesized from 5 micrograms of total RNA using the GeneChip Expression 3′ amplification reagents one-cycle cDNA synthesis kit (Affymetrix), and subsequently column-purified using the GeneChip Sample Cleanup Module. Biotinylated cRNA was synthesized from the double-stranded cDNA by *in vitro* transcription (IVT) using the GeneChip Expression 3′ amplification reagents for IVT labeling (Affymetrix), according to the manufacturer's recommended protocol. Resultant cRNAs were purified by column purification with the GeneChip Sample Cleanup Module (Affymetrix), and quantified. 15 micrograms of cRNA were fragmented by metal-induced hydrolysis in fragmentation buffer (250 mM Tris acetate pH 8.1, 150 mM MgOAc, 500 mM KOAc) at 94°C for 35 minutes. Quality of pre- and post-fragmentation cRNAs was assessed by RNA Nano LabChip analysis on an Agilent Bioanalyzer 2100. Hybridization cocktails were prepared as recommended for arrays of “Standard” format including incubation at 94°C for 5 minutes and 45°C for 5 minutes, and centrifugation at maximum speed for 5 minutes prior to pipetting into the GeneChips (Affymetrix *Plasmodium/Anopheles*). Hybridization was performed at 45°C for 16 hours at 60 rpm in the Affymetrix rotisserie hybridization oven. The signal amplification protocol for washing and staining of eukaryotic targets was performed in an automated fluidics station (Affymetrix FS450). Arrays were scanned in a GeneChip 3000 7G laser scanner with autoloader (Affymetrix) at an emission wavelength of 570 nm and 2.5 µm resolution. Intensity of hybridization for each probe pair was computed by GCOS software.

### Data analysis

Detailed analysis was performed with Genomics Suite Software, version 6.4 (Partek). GC-RMA algorithm defaults were used for background correction (GC-RMA), normalization (Quantile), and summarization (median polish) of probesets. Analysis of variance (ANOVA) was performed with linear contrasts for each *Wolbachia* treatment (strain) vs. control. Gene lists were developed based on 2 fold or greater gene expression and a False Discovery rate P<0.05 criteria. Lists were annotated manually. Immune gene networks were developed using Pathvisio2 [69].

### qPCR verification of expression analysis

Using qPCR, microarray data were validated using infected cell cultures and also somatically-infected mosquitoes. Live female mosquitoes (2 days post emergence) were immobilized on ice and transferred to an electronic cold plate. Mosquitoes were injected with *Wolbachia* (wMelpop or wAlbB) or Schneider's medium as described previously [13]. Although a standard protocol was followed for *Wolbachia* preparations, titers were not explicitly standardized. Injected mosquitoes were incubated at 19°C for 2 days before transfer to 28°C (80% humidity) insectary and were provided with access to a 10% sucrose solution through a cotton wick. After 15 days, mosquitoes were collected and RNA was extracted using TriReagent (Ambion) following manufactures guidelines. For verification of microarray data, total RNA was extracted from Sua5B cell lines (uninfected, wAlbB-infected, or wRi-infected) using the RiboPureTM kit (Ambion) following the manufacturer's instructions. RNA from cells or mosquitoes was DNase treated (Ambion) and cDNA synthesized using superscript III (Invitrogen) following manufactures guidelines. qPCR was performed in triplicate on an AB 7300 Sequence Detection System using the QuantiTect SYBR Green PCR Kit (Qiagen). Analysis was performed using Sequence Detection Software v.1.3 (ABI). Relative quantitation was completed by normalizing gene of interest to the ribosomal protein S7 gene (primers listed in Table 1) and data analyzed using the comparative Ct method (ΔΔCt method) [80].

**Table 1:** List of primers for qPCR.

Affymetrix number	Ensembl number	Description	Primers (5′-3′)
Ag.2L.447.0_CDS_s_at	AGAP005547	Heat shock protein Hsp90	ACGTTACGGGAGACAAG
			ACGATCGATTTGTCCA
Ag.2R.417.0_CDS_a_at	ENSANGG00000013337	Heat shock protein Hsp20	GAGCTGAAGACGGAGTA
			ATCGACGCGACGAGAG
Ag.X.3.0_CDS_at	AGAP000694	Mosquito-specific cecropin	CTTCACCAAGCTGTTCAT
			GCTTGCCGAACTTCC
Ag.2R.818.2_cds_a_at	AGAP004335	Filamin/calponin-like	ACTCTCCGTTCAAGGTTTA
			TTGGCACCGTTCTTAC
Ag.2L.537.2_a_at	AGAP007565	Heat shock protein DnaJ	CGTCAACAAGGACATCG
			ACGGTCCCGTCGAAAT
Ag.2L.2446.0_CDS_at	AGAP005641	Cold-shock DNA-binding domain	ATCGTGCCATGCGTAA
			GGCATTCGGTGTGATA
Ag.2R.20.0_CDS_at	AGAP001377	Serpin	CGGAGATCGAACAGGAT
			ACGAGCGAAACCGTAGT
Ag.3l.42.0_cds_at	AGAP010816	TEP3	CAAACCTCGTTGGTGATA
			GGCGGTGAAATGCTA
Ag.2R.507.1_CDS_a_at	AGAP003696	Aminopeptidase N	TGGTTGGCCGCAGTCAATGGAC
			GGCCGCGAACAGCTTCTCATCAT
Ag.2R.1810.0_CDS_at	AGAP001242	Defective proboscis extension response	ACATACTGACGGTGGGCATTCTC
			CGTTATCCGCAGCGTCCACTCG
Ag.2R.1056.0_CDS_at	AGAP004017	LRR-like	AAATTTGAACCGTCTCGCACATCT
			TAGCCCGTTCACATCGAGTCTTA
Ag.3R.27.0_CDS_a_at	AGAP009212	Serpin6 [54]	CGGTCAGTGGAATCCGGTACTACA
			GCCGTACGCACCATTGGT
Ag.3L.449.0_CDS_at	AGAP011197	FBN9	GAAATTGGCAGTGAGGCGGAGATG
			CCCCTTGTGGTACGTCAGCGAGTC
Ag.3L.13.4_s_at	AGAP011792	CLIP7A	CCTGGACAGCAAGGTGCGGG
			GGAGTTGGAACGCCTCCGGC
	AGAP010592	RP S7 (reference gene) [82]	CATTCTGCCCAAACCGATG
			AACGCGGTCTCTTCTGCTTG

### Accesion numbers

The following is a list of genes and their ENSEMBL or affymetrix accession numbers which are listed in the text: HSP20 AGAP005547, HSP90 Ag.2R.417.0_CDS_a_at, HSPDnaJ AGAP007565 AGAP001810, Cold-shock protein AGAP005641, Cecropin3 AGAP000694, SRPN11 AGAP001377, Filamin, AGAP004335, TEP3 AGAP010816, LRR-like AGAP004017, FBN9 AGAP011197, HSP70 AY137766.1_s_at, PEPCK AGAP003350, Carbonic anhydrase AGAP010052, Laminin AGAP001381 AGAP004993, Collagen AGAP009201, Peroxiredoxin AGAP011824, Superoxide dismutase AGAP010517, glutathione S transferases AGAP004164 AGAP004163 AGAP000761 AGAP009194 AGAP009193 AGAP004173 AGAP000165, CLIP7A AGAP011792, Galectin AGAP012529, CLIPB4 AGAP003250, CLIPB8 AGAP003057, Cecropin1 AGAP000693, TEP15 AGAP008364, GNBPB1 AGAP004455, Caspar AGAP006473, PGRP-LA AGAP005205, Attacin AGAP005620 ANCE AGAP009751 AGAP009756 AGAP009757 AGAP004563 AGAP007622 AGAP004563 AGAP007982, Kazal-like serpin AGAP011482, Crooked neck AGAP001879, Otefin AGAP007603, Dpy-30 AGAP007884, Serac1 AGAP011044, GrpE AGAP011150, ubiquilin AGAP004294, Defective proboscis extension response AGAP001242, Sestrin AGAP007169.

## Supporting Information

Figure S1 Supplementary Figure S1 and associated methods.
(0.06 MB DOC)

Table S1 List of *Anopheles* genes significantly regulated by *Wolbachia* infection.
(0.45 MB XLS)

Table S2 Common *Wolbachia*-regulated genes between *Anopheles* and *Aedes*.
(0.02 MB XLS)

Table S3 Common genes regulated by *Wolbachia* and bacterial challenge in *Anopheles*.
(0.04 MB XLS)
