# Supplementary material for: Wolbachia Infections in Anopheles gambiae Cells: Transcriptomic Characterization of a Novel Host-Symbiont Interaction
Source: PLoS Pathog. 2011 Feb 17;7(2):e1001296. doi: 10.1371/journal.ppat.1001296 (PMC3040664; doi:10.1371/journal.ppat.1001296)
Supplement: Figure S1 — Supplementary Figure S1 and associated methods. (0.06 MB DOC) [file ppat.1001296.s001.doc]

**Supplemental Figure 1. Normalized fold-induction in Sua5B cells after infection by 4 *Rickettsia* species, compared to mock-infected cells. Bars represent median values, error bars represent standard errors. All genes were induced except for gambicin in response to *R. montenensis* infection.**

Supplemental methods:

Rickettsial isolates

*Rickettsia typhi, R. felis, R. montanensis* and *R. peacockii*-infected Sua5B were developed and maintained as described [1].

RNA extraction and reverse transcriptase (RT)-PCR cDNa synthesis

RNA was extracted from *Rickettsia*-infected cells using Trizol (Invitrogen Life Technologies, Carlsbad, CA), purified on RNeasy Mini spin columns (Qiagen Inc, Valencia, CA) and treated with DNase I to eliminate contaminating DNA. Random nonamers were used to prime total RNA and M-MuLV reverse transcriptase was used to synthesize cDNA using the Protoscript II RT-PCR Kit (New England Biolabs, Inc. Ipswich, MA). No-template and no-reverse transcriptase treatments were implemented as negative controls. Primers to Sua5B immune genes are listed in Supplemental Table 4. Quantitative PCR was used to measure the immune response to Rickettsial infection of 4 *Anopheles* immune genes (Cecropin1, Defensin1, Gambicin and Immune-responsive serpin-related protein (IserpF1) in infected Sua5B cells, normalized to S7. Reactions were cinducted in duplicate with 4-5 replicates per treatment as described in the main text. No-template reactions and No-RT reactions served as negative controls. Results are presented as relative fold-change compared to cells mock-infected with cell culture media.

Supplemental Table 4. qPCR Primers used for Rickettsial immune challenge quantification.

| Gene | Primer |
| --- | --- |
| CEC1 | Reference [2] |
|  |  |
| DEF1 | F: CCTTTGTGCCGCTCACTGTATC |
|  | R: ACCCTCTTCCCAGGATGCTAAG |
|  |  |
| Gambicin | Reference [3] |
|  |  |
| IserpF1 | F: ATTGCGACCAACTTCTTCG |
|  | R: TTGACCAGCGTAATGACCG |

Supplemental References

1. Sakamoto JM and Azad AF (2007) Propagation of arthropod-borne Rickettsia spp. in two mosquito cell lines. Applied and Environmental Microbiology 73: 6637-6643.
2. Vizioli J, Bulet P, Charlet M, Lowenberger C, Blass C, Müller HM, Dimopoulos G, Hoffmann J, Kafatos FC, Richman A (2000) Cloning and analysis of a cecropin gene from the malaria vector mosquito, *Anopheles gambiae*. Insect Molecular Biology 9: 75-84.
3. Dong Y, Aguilar R, Xi Z, Warr E, Mongin E, Dimopoulos G (2006) Anopheles gambiae immune responses to human and rodent Plasmodium parasite species. PLoS Pathogens 2: e52.
